# TB Anywhere is TB Everywhere

**DOI:** 10.1186/s13584-018-0233-0

**Published:** 2018-07-20

**Authors:** Jonathan M. Wortham

**Affiliations:** Division of Tuberculosis Elimination, Centers for Disease Control and Prevention, 1600 Clifton Road, Mailstop E-10, Atlanta, Georgia 30329 USA

**Keywords:** Tuberculosis, Infection control, Latent TB infection

## Abstract

To control and prevent outbreaks, public health programs in all countries, regardless of tuberculosis (TB) incidence, must maintain the capacity to perform core control and prevention activities. These include diagnosing and treating cases, contact investigations, and infection prevention and control activities. Congregate settings and healthcare facilities demand special attention, because of the potential for outbreaks associated with infectious cases in these settings. Since almost one-fourth of the world population is thought to be infected with *Mycobacterium tuberculosis,* enhanced efforts to diagnose and treat latent TB infection are needed to prevent future cases and accelerate progress towards TB elimination.

Tuberculosis (TB) anywhere brings the potential for TB everywhere. The outbreak described by Zohar Mor and colleagues in a recent IJHPR article illustrates this principle and its potential consequences [1]. The index patient (i.e., Patient One) in this outbreak was a migrant to Israel from Eritrea, a country with an estimated TB incidence of 74 cases per 100,000 persons, more than 20 times the reported incidence of 3.5 cases per 100,000 persons in Israel [2]. While this patient was presumably, but not definitively, infected with *Mycobacterium tuberculosis* in his birth country, he progressed to infectious TB while living in Israel and working at a long-term care facility. During the subsequent public health investigation, 100 contacts to the index patient in the long-term care facility were identified. Of these, 28 facility residents and 2 staff members had positive tests for TB infection and 1 facility resident had TB disease. During the next year, 2 additional cases were diagnosed in 2 contacts to the index patient. One of these was a facility resident born in the former Soviet Union; former Soviet countries have high TB incidences relative to Israel. TB incidences among former Soviet Countries are between 16 cases per 100,000 persons (Estonia) and 145 cases per 100,000 persons (Kyrgyzstan) [2]. The second was a staff member who experienced TB disease after being diagnosed and treated for latent TB infection (LTBI), but his adherence with LTBI treatment was suboptimal. Along with the evidence from the epidemiologic investigation, the TB program used genotyping data to propose a network of transmission among cases.

To control and prevent these kinds of outbreaks, public health programs in all countries, regardless of TB incidence, must retain capacity to perform core TB control activities [3]. The first, and most fundamental, is to promptly diagnose and treat TB cases. Ensuring universal access to rapid, complete, and effective TB treatment benefits patients, by reducing morbidity, and society, by rendering cases non-infectious and preventing emergence of drug-resistant, more difficult-to-treat forms of disease.

While finding and treating cases might seem straightforward, diagnosing TB is often challenging. Some patients present to healthcare only after they have been contagious for a long time. In 16 of 21 outbreaks in the United States for which the Centers for Disease Control and Prevention (CDC) was invited to provide assistance during 2009–2015, several patients presented to healthcare only after they were contagious for several months [4]. Given the prevalence of homelessness and poverty among patients in these outbreaks, lack of access to routine healthcare likely was a factor behind delayed presentations to healthcare.

Even when patients with TB promptly seek healthcare, they often have non-specific symptoms upon initial presentation. Even medical providers suspect TB, diagnostic tests are not 100% sensitive. As TB incidence declines, clinical expertise for diagnosis and treatment might diminish, because fewer clinicians encounter TB cases during training and practice. Prolonged infectiousness due, at least in part, to misdiagnosis is relatively common in the United States, despite widespread availability of diagnostic tests for TB infection and disease. In two-thirds of outbreaks in the United States for which CDC provided assistance, many patients presented to healthcare multiple times and received other diagnoses and treatments before their TB diagnoses were made [4]. In all, delayed diagnoses, whether due to delayed healthcare seeking or initial misdiagnoses after healthcare seeking, contributed to all but one CDC-investigated outbreak during 2009–2015. Because cases were infectious for such long periods, these outbreaks involved more infectious cases to be treated and contacts to be evaluated, resulting in longer, more resource-intensive public health responses.

Making prompt TB diagnoses among healthcare workers and persons living or working in congregate settings (e.g., homeless overnight facilities) deserves special attention because of the consequences of contagious persons in these settings. In the United States, healthcare facilities, correctional facilities, and homeless overnight facilities should have specific infection control procedures designed to prevent *M. tuberculosis* transmission [5–7]. Administrative measures designed to reduce the risk for exposure to persons who might have TB disease, are foundational to any TB infection control policy. In order to prevent facility-associated transmission, healthcare facilities should immediately exclude workers experiencing symptoms consistent with TB from patient care activities until they are determined not to have infectious TB. Similarly, symptomatic persons should be excluded from congregate settings (e.g., homeless overnight facilities), unless effective airborne infection isolation can be maintained, until they are determined not to have TB or until they are determined to be not contagious [4, 5].

Other types of controls supplement administrative efforts to reduce exposure to persons with contagious TB. Environmental controls, such as high efficiency particulate air (HEPA) filtration, to reduce the concentration of infectious droplet nuclei in air, and respiratory-protection controls, such as fit-tested N95 masks, are second and third in the hierarchy of TB infection control activities and can provide additional layers of protection [5]. Annual TB testing programs for healthcare workers, when appropriate, also facilitate surveillance for *M. tuberculosis* transmission in these settings [5].

To facilitate prompt diagnoses among immigrants, some countries administer screening programs similar to the radiograph-based program at the Israeli-Egyptian border described by Mor and colleagues. The United States’ immigration requirements for adults include a medical history, physical examination, and chest radiograph. Additionally, microscopic examination of three sputum smears and cultures are required for persons who have any clinical symptoms or radiographic signs of TB or HIV infection. Persons treated for TB disease and those who have smear- or culture-positive disease must complete treatment before immigration [8]. While implementation of these programs facilitates identification of immigrants with disease, these programs are not, in their current forms, specifically designed to identify or treat asymptomatic persons with latent TB infection (LTBI). Presumably, the index patient in the outbreak that Mor and colleagues describe had LTBI when he immigrated and progressed to infectious, pulmonary TB disease later. If he had LTBI and was diagnosed and treated before he developed disease, this outbreak might have been prevented. Identifying and treating LTBI will be important to further reduce TB burden, because the majority of TB cases in the United States occur among persons born in other countries and likely represent reactivation of infections acquired outside of the United States, including an increasing proportion of persons diagnosed >10 years after U.S. arrival [9].

Even though the majority of U.S. TB cases likely represent reactivation of LTBI, public health measures to control and prevent *M. tuberculosis* transmission remain important. Contact investigations facilitate identification and treatment of TB disease and LTBI among persons exposed to infectious cases; effective contact investigations help interrupt transmission through facilitating TB and LTBI diagnoses before patients can become contagious and spread disease to others. However, these investigations can be challenging if patients are unwilling or unable to name contacts. In the United States, universal genotyping of isolates from culture-confirmed cases facilitates outbreak detection among TB patients who could not or would not name contacts. Analyses of genotyping data facilitated detection of the majority of outbreaks CDC has investigated since 2009 [4]; many of these outbreaks occurred in congregate settings, such as homeless overnight facilities, where patients were not able to name their contacts. Detection and investigation of these outbreaks prompted targeted efforts to interrupt transmission, such as strengthening of infection control and prevention policies at homeless overnight facilities. Additionally, through reviews of overnight facility records, public health personnel investigators were able to identify and evaluate contacts for TB disease and LTBI.

Traditionally, as with Patient Three in the outbreak described by Mor, contacts with LTBI received 9 months’ of daily, self-administered treatment with isoniazid to prevent TB disease. While efficacious under trial conditions, non-adherence with the treatment regimen, at least in part because of the long duration of therapy, limits the real-world effectiveness of the regimen for preventing TB cases. More recently, however, shorter course regimens for LTBI treatment have been introduced, including 4 months’ of daily rifampin or isoniazid and rifapentine given once weekly for 12 weeks [10]. The efficacy of these shorter course regimens is comparable to isoniazid, but treatment completion is superior. Since so many people – as much as one-fourth of the world’s population – are latently infected with *M. tuberculosis,* expansion of LTBI diagnosis and treatment beyond contacts will be critical in order to make progress towards TB elimination.

Since 2016, the United States Preventive Services Task Force, a group charged with making recommendations about the effectiveness of specific preventive care services for patients without obvious related signs or symptoms, has recommended testing “populations at risk for LTBI” in the United States. This includes persons born in high-incidence countries as well as persons living in “high-risk” congregate settings, including long-term care facilities and homeless shelters [11]. Along with efforts to develop more sensitive and specific diagnostic tests for LTBI and shorter treatment regimens to optimize adherence and effectiveness, targeted efforts to increase implementation of these recommendations are needed to prevent more TB cases among patients like the index patient in this outbreak and accelerate progress towards TB elimination.
